# Imidazolium *trans*-di­aqua­dioxalato­chromate(III) dihydrate

**DOI:** 10.1107/S160053681303078X

**Published:** 2013-11-16

**Authors:** Ichraf Chérif, Jawher Abdelhak, Mohamed Faouzi Zid, Ahmed Driss

**Affiliations:** a Laboratoire de Matériaux et Cristallochimie, Faculté des Sciences de Tunis, Université de Tunis El Manar, 2092 Manar II Tunis, Tunisia

## Abstract

In the title hydrated mol­ecular salt, (C_3_H_5_N_2_)[Cr(C_2_O_4_)_2_(H_2_O)_2_]·2H_2_O, the complete cation is generated by a crystallographic twofold rotation axis, with one C atom lying on the rotation axis. The complete anion is generated by crystallographic inversion symmetry (Cr^III^ site symmetry -1), to generate a slightly distorted CrO_6_ octa­hedron with *trans* water mol­ecules and chelating oxalate dianions. The oxalate ion is almost planar (r.m.s. deviation = 0.017 Å) and the five-membered chelate ring is a shallow envelope with the metal ion displaced by 0.126 (1) Å from the ligand atoms. The crystal structure features O—H⋯O, N—H⋯O and C—H⋯O hydrogen bonds, which link the components into a three-dimensional network.

## Related literature

For a related structure and background to oxalate complexes, see: Chérif *et al.* (2012 ▶). For the structures of salts containing the [Cr(C_2_O_4_)_2_(H_2_O)_2_]^−^ anion with various cations, see: Bélombé *et al.* (2009 ▶); Nenwa *et al.* (2010 ▶); Chérif *et al.* (2011 ▶); Kahlenberg *et al.* (2011 ▶). For geometric parameters of the imidazolium cation, see: Zhu (2012 ▶); Smith & Wermuth (2010 ▶).
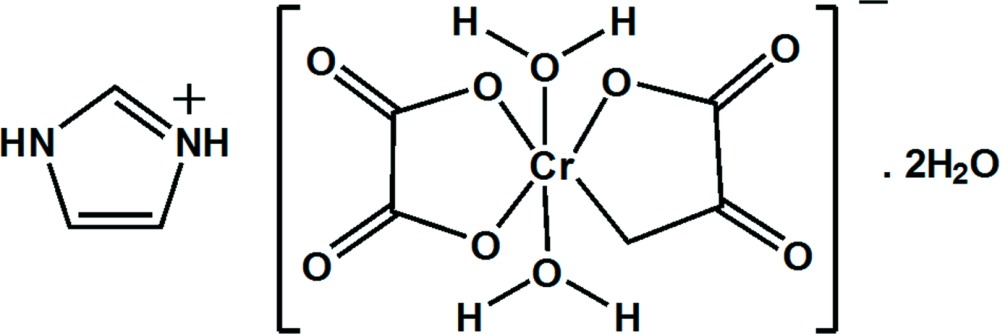


## Experimental

### 

#### Crystal data

(C_3_H_5_N_2_)[Cr(C_2_O_4_)_2_(H_2_O)_2_]·2H_2_O
*M* *_r_* = 369.19
Monoclinic, 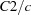

*a* = 10.836 (1) Å
*b* = 7.5409 (7) Å
*c* = 16.349 (3) Å
β = 93.52 (1)°
*V* = 1333.4 (3) Å^3^
*Z* = 4
Mo *K*α radiation
μ = 0.93 mm^−1^
*T* = 298 K
0.6 × 0.4 × 0.3 mm

#### Data collection

Enraf–Nonius CAD-4 diffractometer
Absorption correction: ψ scan (North *et al.*, 1968 ▶) *T*_min_ = 0.647, *T*_max_ = 0.757
1848 measured reflections
1452 independent reflections
1369 reflections with *I* > 2σ(*I*)
*R*_int_ = 0.011
2 standard reflections every 120 min intensity decay: 2.2%

#### Refinement

*R*[*F* ^2^ > 2σ(*F* ^2^)] = 0.029
*wR*(*F* ^2^) = 0.086
*S* = 1.12
1452 reflections
124 parameters
H atoms treated by a mixture of independent and constrained refinement
Δρ_max_ = 0.55 e Å^−3^
Δρ_min_ = −0.39 e Å^−3^

### 

Data collection: *CAD-4 EXPRESS* (Duisenberg, 1992 ▶; Macíček & Yordanov, 1992 ▶); cell refinement: *CAD-4 EXPRESS*; data reduction: *XCAD4* (Harms & Wocadlo, 1995 ▶); program(s) used to solve structure: *SHELXS97* (Sheldrick, 2008 ▶); program(s) used to refine structure: *SHELXL97* (Sheldrick, 2008 ▶); molecular graphics: *DIAMOND* (Brandenburg & Putz, 1999 ▶); software used to prepare material for publication: *WinGX* (Farrugia, 2012 ▶).

## Supplementary Material

Crystal structure: contains datablock(s) I, global. DOI: 10.1107/S160053681303078X/hb7157sup1.cif

Structure factors: contains datablock(s) I. DOI: 10.1107/S160053681303078X/hb7157Isup2.hkl

Additional supplementary materials:  crystallographic information; 3D view; checkCIF report

## Figures and Tables

**Table 1:** Selected bond lengths (Å)

Cr—O3	1.963 (1)
Cr—O2	1.967 (1)
Cr—O1	1.979 (2)

**Table 2:** Hydrogen-bond geometry (Å, °)

*D*—H⋯*A*	*D*—H	H⋯*A*	*D*⋯*A*	*D*—H⋯*A*
O6—H1⋯O4^i^	0.80 (3)	2.20 (3)	2.984 (2)	168 (3)
O6—H4⋯O4^ii^	0.72 (3)	2.16 (3)	2.878 (2)	174 (4)
O1—H2⋯O5^iii^	0.79 (3)	1.93 (3)	2.717 (2)	173 (3)
O1—H3⋯O6^iv^	0.85 (3)	1.75 (3)	2.601 (2)	176 (3)
N1—H5⋯O3	1.04 (4)	2.07 (3)	2.926 (2)	137 (2)
C3—H7⋯O2^iv^	0.93	2.25	3.088 (3)	150

Symmetry codes: (i) 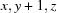; (ii) 
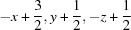
; (iii) 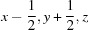; (iv) 
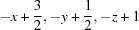
.

**Fig. 1.**
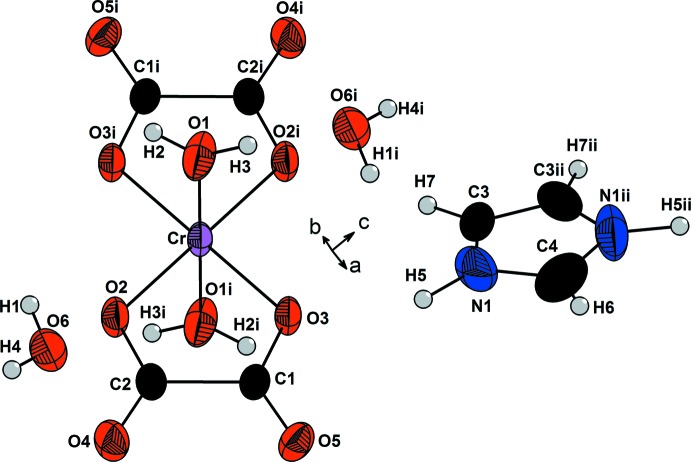
: A view of the title compound with displacement ellipsoids drawn at the 50% probability level for non-H atoms. [Symmetry codes: (i) -x + 3/2,-y + 1/2,-z + 1; (ii) -x + 2,y,-z + 3/2].

**Fig. 2.**
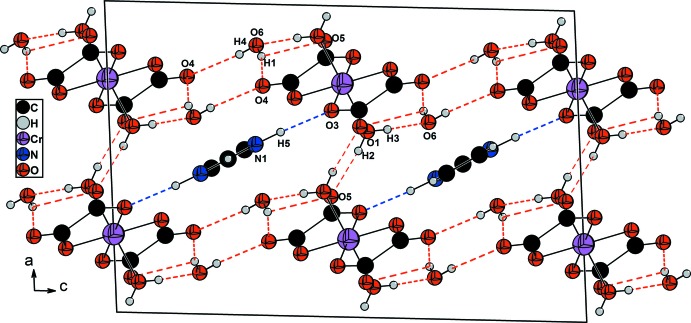
: Structure projection along b axis showing O—H···O (red dashed lines) and N—H···O (blue dashed lines) hydrogen bonds.

## supplementary crystallographic information

### 1. Comment

As part of our ongoing studies of new bis(oxalato)chromate(III) species of general formula (organic cation)[Cr(C_2_O_4_)_2_(H_2_O)_2_].nH_2_O (Chérif *et al.*, 2012), we now describe the synthesis and structure of the title compound, (I).

The asymmetric unit of (I) is formed by one-half cation, one half anion and one water molecule of crystallization (Fig. 1). The Cr^+III^ ion lies on an inversion center and the C and H atoms of C(4)—H(6) groups lie on twofold rotation axis. In the anionic complex, the coordination environment of Cr^+III^ ion involves six oxygen atoms (two from *trans* water molecules and four from two chelating oxalate dianion) in a slightly distorted octahedral geometry. The main distortion of the CrO_6_ octahedron is associated to the reduction from the ideal 90° value of some bond angles [83.11 (5)° for O(3)—Cr—O(2) and O(3)^i^—Cr—O(2)^i^]. The equatorial Cr—O_(ox)_ distances are very similar, 1.963 (1) Å [Cr—O(3), Cr—O(3)^i^] and 1.967 (1) Å [Cr—O(2), Cr—O(2)^i^], and they are comparable with the values reported for similar compounds containing the [Cr(C_2_O_4_)_2_(H_2_O)_2_]^-^ motif completed with various uncoordinated cations including quinolinium: [C_9_H_8_N][Cr(H_2_O)_2_(C_2_O_4_)_2_] (Bélombé *et al.*, 2009), 4-dimethylaminopyridinium: [C_7_H_11_N_2_][Cr(C_2_O_4_)_2_(H_2_O)_2_] (Nenwa *et al.*, 2010), 4-aminopyridinium: [C_5_H_7_N_2_][Cr(C_2_O_4_)_2_(H_2_O)_2_]·H_2_O (Chérif *et al.*, 2011), 1-ethyl-3-methylimidazolium: [EMIm][Cr(C_2_O_4_)_2_(H_2_O)_2_] (Kahlenberg *et al.*, 2011) and 3-aminopyridinium (C_5_H_7_N_2_)[Cr(C_2_O_4_)_2_(H_2_O)_2_] (Chérif *et al.*, 2012). The axial Cr—O_(water)_ distances of 1.979 (2) Å are somewhat longer than the Cr—O_(ox)_ ones but significantly shorter than those for compounds already mentioned. As far as the imidazolium cations are concerned, the C—N [1.363 (3) Å for N(1)—C(4) and N(1)^ii^—C(4), 1.295 (3) Å for N(1)—C(3) and N(1)^ii^—C(3)^ii^] and C—C bond lengths of 1.315 (4) Å for C(3)—C(3)^ii^, agree with those reported for similar compounds (Zhu, 2012; Smith & Wermuth, 2010).

Within the crystal structure, hydrogen bonds consisting of O—H···O, N—H···O and C—H···O interactions contribute to the cohesion of the packing (Fig. 2). In fact, for the O—H···O hydrogen bonds, the uncoordinated water molecules [O(6)] play a role as both acceptors and donors while the coordinated one [O(1)] act only as donors. Finally, the [Cr(C_2_O_4_)_2_(H_2_O)_2_]^-^ anions and (C_3_H_5_N_2_)^+^ cations are linked through N(1)—H(5)···O(3) and C(3)—H(7)···O(2) interactions. As a consequence, the overall hydrogen-bonded scheme can be described as a three-dimensional network.

### 2. Experimental

A mixture of imidazole (1 mmol), oxalic acid dihydrate (2 mmol) and Cr(NO_3_)_3_·9H_2_O (1 mmol) was dissolved in 40 ml of water. The resulting solution was then stirred for 2 h and allowed to evaporate at room temperature. After two months, violet prisms of (I) were obtained.

### 3. Refinement

All non hydrogen atoms were treated anisotropically and the H atoms were refined isotropically. After several cycles of refinement, the hydrogen atoms were found in a difference Fourier map and the H(7) was placed in calculated position with C—H distance of 0.93 Å and constrained to ride on his parent atom [C(3)] with *U*_iso_(H)=1.2*U*_eq_(C).

### Crystal data

**Table:**

(C_3_H_5_N_2_)[Cr(C_2_O_4_)_2_(H_2_O)_2_]·2H_2_O	*F*(000) = 756
*M**_r_* = 369.19	*D*_x_ = 1.839 Mg m^−^^3^
Monoclinic, *C*2/*c*	Mo *K*α radiation, λ = 0.71073 Å
Hall symbol: -C 2yc	Cell parameters from 25 reflections
*a* = 10.836 (1) Å	θ = 10–15°
*b* = 7.5409 (7) Å	µ = 0.93 mm^−^^1^
*c* = 16.349 (3) Å	*T* = 298 K
β = 93.52 (1)°	Prism, violet
*V* = 1333.4 (3) Å^3^	0.6 × 0.4 × 0.3 mm
*Z* = 4	

### Data collection

**Table:**

Enraf–Nonius CAD-4 diffractometer	1369 reflections with *I* > 2σ(*I*)
Radiation source: fine-focus sealed tube	*R*_int_ = 0.011
Graphite monochromator	θ_max_ = 27.0°, θ_min_ = 2.5°
ω/2θ scans	*h* = −13→13
Absorption correction: ψ scan (North *et al.*, 1968)	*k* = −1→9
*T*_min_ = 0.647, *T*_max_ = 0.757	*l* = −20→1
1848 measured reflections	2 standard reflections every 120 min
1452 independent reflections	intensity decay: 2.2%

### Refinement

**Table:**

Refinement on *F*^2^	Primary atom site location: structure-invariant direct methods
Least-squares matrix: full	Secondary atom site location: difference Fourier map
*R*[*F*^2^ > 2σ(*F*^2^)] = 0.029	Hydrogen site location: inferred from neighbouring sites
*wR*(*F*^2^) = 0.086	H atoms treated by a mixture of independent and constrained refinement
*S* = 1.12	*w* = 1/[σ^2^(*F*_o_^2^) + (0.0456*P*)^2^ + 1.7165*P*] where *P* = (*F*_o_^2^ + 2*F*_c_^2^)/3
1452 reflections	(Δ/σ)_max_ < 0.001
124 parameters	Δρ_max_ = 0.55 e Å^−^^3^
0 restraints	Δρ_min_ = −0.39 e Å^−^^3^

### Special details

**Table:**

Geometry. All e.s.d.'s (except the e.s.d. in the dihedral angle between two l.s. planes) are estimated using the full covariance matrix. The cell e.s.d.'s are taken into account individually in the estimation of e.s.d.'s in distances, angles and torsion angles; correlations between e.s.d.'s in cell parameters are only used when they are defined by crystal symmetry. An approximate (isotropic) treatment of cell e.s.d.'s is used for estimating e.s.d.'s involving l.s. planes.
Refinement. Refinement of *F*^2^ against ALL reflections. The weighted *R*-factor *wR* and goodness of fit *S* are based on *F*^2^, conventional *R*-factors *R* are based on *F*, with *F* set to zero for negative *F*^2^. The threshold expression of *F*^2^ > σ(*F*^2^) is used only for calculating *R*-factors(gt) *etc*. and is not relevant to the choice of reflections for refinement. *R*-factors based on *F*^2^ are statistically about twice as large as those based on *F*, and *R*- factors based on ALL data will be even larger.

### Fractional atomic coordinates and isotropic or equivalent isotropic displacement parameters (Å^2^)

**Table:**

	*x*	*y*	*z*	*U*_iso_*/*U*_eq_	
Cr	0.7500	0.2500	0.5000	0.02258 (15)	
O2	0.70111 (13)	0.12749 (18)	0.39687 (8)	0.0324 (3)	
O3	0.83676 (12)	0.02646 (17)	0.52566 (8)	0.0276 (3)	
O4	0.73747 (15)	−0.12361 (19)	0.33002 (9)	0.0387 (4)	
O6	0.86780 (17)	0.5293 (2)	0.31972 (10)	0.0402 (4)	
C1	0.83297 (15)	−0.0860 (2)	0.46642 (11)	0.0242 (4)	
O1	0.59861 (14)	0.1644 (2)	0.54913 (11)	0.0424 (4)	
O5	0.88681 (13)	−0.22844 (18)	0.46604 (9)	0.0334 (3)	
C2	0.75010 (16)	−0.0264 (2)	0.38993 (11)	0.0264 (4)	
C3	0.9690 (2)	0.1333 (3)	0.71415 (13)	0.0372 (5)	
H7	0.9425	0.2337	0.6851	0.045*	
N1	0.95088 (19)	−0.0291 (4)	0.69055 (13)	0.0575 (6)	
C4	1.0000	−0.1396 (5)	0.7500	0.0518 (9)	
H1	0.832 (3)	0.618 (4)	0.3298 (18)	0.051 (8)*	
H2	0.535 (3)	0.199 (4)	0.5285 (19)	0.055 (8)*	
H3	0.606 (3)	0.101 (4)	0.592 (2)	0.056 (8)*	
H4	0.837 (3)	0.493 (5)	0.283 (2)	0.072 (12)*	
H5	0.910 (4)	−0.078 (5)	0.636 (2)	0.085 (11)*	
H6	1.0000	−0.257 (7)	0.7500	0.087 (18)*	

### Atomic displacement parameters (Å^2^)

**Table:**

	*U*^11^	*U*^22^	*U*^33^	*U*^12^	*U*^13^	*U*^23^
Cr	0.0263 (2)	0.0204 (2)	0.0200 (2)	0.00213 (14)	−0.00693 (15)	0.00055 (13)
O2	0.0411 (7)	0.0286 (7)	0.0257 (6)	0.0078 (6)	−0.0137 (5)	−0.0039 (5)
O3	0.0326 (7)	0.0246 (6)	0.0242 (6)	0.0035 (5)	−0.0095 (5)	0.0005 (5)
O4	0.0485 (8)	0.0352 (8)	0.0306 (7)	0.0058 (6)	−0.0109 (6)	−0.0097 (6)
O6	0.0554 (10)	0.0333 (8)	0.0311 (8)	0.0038 (7)	−0.0030 (7)	−0.0006 (6)
C1	0.0222 (8)	0.0235 (8)	0.0262 (8)	−0.0013 (6)	−0.0029 (6)	0.0014 (7)
O1	0.0296 (8)	0.0528 (10)	0.0441 (9)	0.0015 (7)	−0.0030 (7)	0.0219 (8)
O5	0.0326 (7)	0.0260 (7)	0.0408 (8)	0.0062 (5)	−0.0053 (6)	−0.0010 (6)
C2	0.0277 (8)	0.0267 (9)	0.0242 (8)	−0.0009 (7)	−0.0045 (7)	−0.0010 (7)
C3	0.0401 (11)	0.0382 (11)	0.0335 (10)	0.0124 (9)	0.0040 (8)	0.0118 (9)
N1	0.0402 (10)	0.0946 (19)	0.0362 (10)	0.0027 (11)	−0.0099 (8)	−0.0193 (11)
C4	0.0457 (18)	0.0294 (16)	0.080 (3)	0.000	0.0024 (17)	0.000

### Geometric parameters (Å, º)

**Table:**

Cr—O3^i^	1.963 (1)	C1—O5	1.223 (2)
Cr—O3	1.963 (1)	C1—C2	1.560 (2)
Cr—O2	1.967 (1)	O1—H2	0.79 (4)
Cr—O2^i^	1.967 (1)	O1—H3	0.85 (3)
Cr—O1^i^	1.979 (2)	C3—N1	1.295 (3)
Cr—O1	1.979 (2)	C3—C3^ii^	1.315 (4)
O2—C2	1.284 (2)	C3—H7	0.9300
O3—C1	1.286 (2)	N1—C4	1.363 (3)
O4—C2	1.224 (2)	N1—H5	1.04 (4)
O6—H1	0.80 (3)	C4—N1^ii^	1.363 (3)
O6—H4	0.73 (4)	C4—H6	0.89 (5)
			
O3^i^—Cr—O3	180.0	O5—C1—O3	126.15 (16)
O3^i^—Cr—O2	96.89 (5)	O5—C1—C2	120.02 (16)
O3—Cr—O2	83.11 (5)	O3—C1—C2	113.83 (14)
O3^i^—Cr—O2^i^	83.11 (5)	Cr—O1—H2	116 (2)
O3—Cr—O2^i^	96.89 (5)	Cr—O1—H3	119 (2)
O2—Cr—O2^i^	180.0	H2—O1—H3	125 (3)
O3^i^—Cr—O1^i^	91.78 (7)	O4—C2—O2	125.83 (17)
O3—Cr—O1^i^	88.22 (7)	O4—C2—C1	119.95 (16)
O2—Cr—O1^i^	89.52 (7)	O2—C2—C1	114.21 (15)
O2^i^—Cr—O1^i^	90.48 (7)	N1—C3—C3^ii^	108.99 (13)
O3^i^—Cr—O1	88.22 (7)	N1—C3—H7	125.5
O3—Cr—O1	91.78 (7)	C3^ii^—C3—H7	125.5
O2—Cr—O1	90.48 (7)	C3—N1—C4	108.7 (2)
O2^i^—Cr—O1	89.52 (7)	C3—N1—H5	130 (2)
O1^i^—Cr—O1	180.0	C4—N1—H5	122 (2)
C2—O2—Cr	114.16 (11)	N1^ii^—C4—N1	104.7 (3)
C1—O3—Cr	114.36 (11)	N1^ii^—C4—H6	127.67 (16)
H1—O6—H4	107 (3)	N1—C4—H6	127.67 (15)

Symmetry codes: (i) −*x*+3/2, −*y*+1/2, −*z*+1; (ii) −*x*+2, *y*, −*z*+3/2.

### Hydrogen-bond geometry (Å, º)

**Table:**

*D*—H···*A*	*D*—H	H···*A*	*D*···*A*	*D*—H···*A*
O6—H1···O4^iii^	0.80 (3)	2.20 (3)	2.984 (2)	168 (3)
O6—H4···O4^iv^	0.72 (3)	2.16 (3)	2.878 (2)	174 (4)
O1—H2···O5^v^	0.79 (3)	1.93 (3)	2.717 (2)	173 (3)
O1—H3···O6^i^	0.85 (3)	1.75 (3)	2.601 (2)	176 (3)
N1—H5···O3	1.04 (4)	2.07 (3)	2.926 (2)	137 (2)
C3—H7···O2^i^	0.93	2.25	3.088 (3)	150

Symmetry codes: (i) −*x*+3/2, −*y*+1/2, −*z*+1; (iii) *x*, *y*+1, *z*; (iv) −*x*+3/2, *y*+1/2, −*z*+1/2; (v) *x*−1/2, *y*+1/2, *z*.
